# The Recombinant Expression Proteins FnBP and ClfA From *Staphylococcus aureus* in Addition to GapC and Sip From *Streptococcus agalactiae* Can Protect BALB/c Mice From Bacterial Infection

**DOI:** 10.3389/fvets.2021.666098

**Published:** 2021-06-24

**Authors:** Zhongchen Ma, Xinyue Yin, Peng Wu, Ruirui Hu, Yong Wang, Jihai Yi, Zhen Wang, Chuangfu Chen

**Affiliations:** ^1^International Joint Research Center for Animal Health Breeding, College of Animal Science and Technology, Shihezi University, Shihezi, China; ^2^Collaborative Innovation Center for Prevention and Control of High Incidence Zoonotic Infectious Diseases in Western China, College of Animal Science and Technology, Shihezi University, Shihezi, China; ^3^College of Life Sciences, Shihezi University, Shihezi, China

**Keywords:** *Staphylococcus aureus*, *Streptococcus agalactiae*, FnBP+ClfA+GapC+Sip gene, recombinant fusion expression, challenge protection experiment

## Abstract

Dairy cow mastitis is a serious disease that is mainly caused by intramammary infection with *Staphylococcus aureus* and *Streptococcus agalactiae* [group B streptococcus (GBS)]. FnBP and ClfA are the virulence factors of *S. aureus*, while GapC is the respective factor for *S. agalactiae*. Sip is a highly immunogenic protein, and it is conserved in all GBS serotypes. In this study, we analyzed the abovementioned four genes prepared a FnBP+ClfA chimeric protein (FC), a GapC+Sip chimeric protein (GS), and a FnBP+ClfA+GapC+Sip chimeric protein (FCGS) based on the antigenic sites to evaluate their use in vaccine development. After expression and purification of the recombinant proteins in *Escherichia coli*, BALB/c mice were immunized with them to examine resistance effects. The total lethal and half lethal doses of *S. aureus* and *S. agalactiae* were then measured, and the immunoprotective effects of the fusion proteins were evaluated. The FC and FCGS chimeric proteins could induce mice to produce high levels of antibodies, and bacterial loads were significantly reduced in the spleens and livers after challenge. After immunization with FCGS, the recipients resisted the attacks of both *S. aureus* and *S. agalactiae*, indicating the potential of the fusion protein as a mastitis vaccine.

## Introduction

Mastitis is a disease of dairy cows that causes significant economic losses in the dairy farming industry (1). Pathogenic microbial infection as well as physical and chemical damage can lead to dairy cow mastitis, although infection by pathogenic microorganisms is the main cause (2). Pathogens associated with mastitis include *Staphylococcus aureus* and *Streptococcus agalactiae* that both cause intramammary infections (IMIs) and thereby increase the difficulty of treating cow mastitis (3, 4).

Many studies have shown that *S. aureus* and *S. agalactiae* are the most common pathogens causing chronic, infectious, and refractory bovine mastitis (5, 6). Further, the infection prevalence of *S. aureus* in Chinese dairy herds is 29% (7), which is significantly higher than in western country herds (8). There is currently no effective vaccine for dairy cow mastitis, and the treatment of dairy cow mastitis is becoming increasingly more difficult with the emergence of bacterial resistance to treatment (9).

Bacterial adhesins including fibronectin-binding protein (FnBP) and aggregation factor A (ClfA) play key roles in pathogenic invasion (10). FnBP and ClfA are the two surface adhesins of *S. aureus*. ClfA binds to complement factor I and is an important factor that promotes evasion of neutrophil-related death (11). FnBP and elastin (12) mainly bind to plasminogen (13) and promote biofilm formation. Previous studies have shown that antibodies induced by FnBPs and ClfA can partially block the adhesion of *S. aureus* to breast tissue (14, 15). In addition, the mixed bivalent nucleic acid vaccine for *S. aureus* that targets FnBPs and ClfA (16) can effectively stimulate specific immune responses in dairy cows and the immunoprotective effects of an FnBP+fusion protein has been confirmed in mice (17).

Surface immunogenic protein (Sip) is an important adhesion factor on the surface of *S. agalactiae*. It is a highly conserved protein that is expressed in all *S. agalactiae* serotypes, and the protein can induce cross-immunity protection (18). In addition, GapC protein is a streptococcal surface dehydrogenase (SDH) with glyceraldehyde 3-phosphate dehydrogenase (DAPDH) activity (19). GapC exhibits considerable homology at the DNA and amino acid levels to homologs among different *S. agalactiae* strains (20). The protein is known to play an important role in the pathogenesis of dairy cow mastitis, and the use of GapC protein to immunize dairy cows yields significant immune protection (21). Consequently, GapC protein is an important target for cow mastitis vaccines (21, 22). However, the joint use of FnBP and ClfA from *S. aureus* in addition to GapC and Sip of *S. agalactiae* has rarely been studied, and no safe and effective mastitis vaccine has been reported.

Consequently, the goal of this study was to combine FnBP and ClfA of *S. aureus* with GapC and Sip of *S. agalactiae* to develop candidate recombinant proteins with activity against *S. aureus* and *S. agalactiae* infection. The recombinant proteins could then be used to further develop a cow mastitis vaccine. Specifically, the fusion proteins FnBP+ClfA (FC), GapC+Sip (GS), and FnBP+ClfA+GapC+Sip (FCGS) were constructed; and the effects of the fusion proteins on *S. aureus* and *S. agalactiae* infections were analyzed in mice. The results demonstrated that FC, GS, and FCGS can be used as potential vaccine proteins and are important targets for further research.

## Materials and Methods

### Ethical Approval

All animals were treated humanely and in accordance with institutional animal care guidelines. This study was approved by the Animal Care and Use Committee of Shihezi University.

### Strains and Animals

Standard *S. aureus* [American Type Culture Collection (ATCC) 25923] and *S. agalactiae* (ATCC 13813) strains were provided by the ATCC and cultured in brain heart infusion (BHI) broth/agar (Hopebio, China) at 37°C. In addition, *Escherichia coli* strains DH5α (Sigma-Aldrich Corp., St. Louis, MO, USA) and C43 (DE3, Sigma, USA) were cultured in Luria–Bertani medium (Difco, Becton Dickinson, Franklin Lakes, New Jersey, USA). Five-week-old female BALB/c mice were purchased from the Experimental Animal Center of the Academy of Military Medical Science (Beijing, China). All experimental procedures and animal care protocols were performed in compliance with institutional animal care regulations.

### Acquisition of FC, GS, and FCGS Gene Sequences and Bioinformatics Analysis

FnBP (gene ID: DQ447162) and ClfA (gene ID: EF207779) gene sequences from *S. aureus* in addition to GapC (gene ID: af421899) and Sip (gene ID: fj808732) gene sequences of *S. agalactiae* were retrieved from the GenBank database. The Kolaskar and Tongaonkar methods were used to predict and analyze the B cell epitopes of FnBP, ClfA, GapC, and Sip gene sequences (http://imed.med.ucm.es/Tools/antigenic.pl) (23). The SignalP 5.0 Server (http://www.cbs.dtu.dk/services/SignalP/) (24, 25) was used to predict the presence of signal peptides in the proteins; and fragments with excellent immunogenicity were selected and divided into FC, GS, and FCGS combination groups by adding a linker sequence (-GGGGSGGGGSGGGGS-) to tightly combine FC, GS, and FCGS. After the target genes were optimized, we commissioned GENERAL BIOL (Anhui, China) to synthesize the sequences and constructed each tandem sequence (FC, GS, and FCGS) by ligation into the vector pET28a/pET32a (EMD Biosciences, Novagen, San Diego, CA, USA). The Phyre2 software program (http://www.sbg.bio.ic.ac.uk/phyre2/html/page.cgi?id=index) was used to predict the tertiary structures of the recombinant fusion proteins (26).

### Expression and Identification of FC, GS, and FCGS Proteins

Isopropyl β-d-1-thiogalactopyranoside (IPTG; Solarbio, Beijing, China) was used to induce recombinant expression in the strains pET32a-FC-DE3, pET28a-GS-DE3, and pET32a-FCGS-DE3. After cells were collected, sodium dodecyl sulfate–polyacrylamide gel electrophoresis (SDS-PAGE) was used to analyze the soluble expression of the target proteins. Target protein purification was performed with a His-Tagged Protein Purification Kit using an inclusion body protein (CWBIO, Beijing, China).

Target protein concentrations were quantified with a Micro BCA Protein Assay Kit (Thermo Fisher Scientific, Waltham, MA, USA), and Western blotting analysis was used to verify the reactogenicity of the purified proteins. The primary antibody used positive *S. aureus*/*S. agalactiae* antibody bovine serum (1:100) that was provided by International Joint Research Center for Animal Health Breeding (Shihezi University) (unpublished). The secondary antibody comprised rabbit anti-bovine IgG/horseradish peroxidase (HRP) (1:2,000; Solarbio, China). A SuperSignal West Femto Trial kit (Thermo, USA) was then used to develop color.

### Determination of Lethal Bacterial Dosages in BALB/c Mice

The total lethal levels of *S. aureus* and *S. agalactiae* toward BALB/c mice were first determined. *S. aureus* was cultivated to the order of 2.1 × 10^10^ CFU/ml. BALB/c mice were divided into six groups that were intraperitoneally inoculated with 5 μl (1.05 × 10^8^ CFU/mouse), 10 μl (2.1 × 10^8^ CFU/mouse), 15 μl (3.15 × 10^8^ CFU/mouse), 20 μl (4.2 × 10^8^ CFU/mouse), 200 μl (4.2 × 10^9^ CFU/mouse), and 400 μl (8.4 × 10^9^ CFU/mouse) of culture. *S. agalactiae* was also cultivated to 3.3 × 10^10^ CFU/ml, and BALB/c mice were divided into six groups that were intraperitoneally injected with 50 μl (1.65 × 10^9^ CFU/mouse), 100 μl (3.3 × 10^9^ CFU/mouse), 150 μl (4.95 × 10^9^ CFU/mouse), 200 μl (6.6 × 10^9^ CFU/mouse), and 400 μl (1.32 × 10^10^ CFU/mouse) of *S*. *agalactiae* culture. Six mice were immunized with each dose, and the clinical manifestations and time of death for mice were observed and recorded for seven consecutive days.

The median lethal doses of *S. aureus* and *S. agalactiae* toward BALB/c mice were then determined. In these experiments, BALB/c mice were divided into six groups, and each group comprised six mice that were intraperitoneally injected with 16 μl (3.36 × 10^8^ CFU/mouse), 18 μl (3.9 × 10^8^ CFU/mouse), and 20 μl (4.2 × 10^8^ CFU/mouse) of *S. aureus* culture. In addition, BALB/c mice were divided into six groups, and six mice in each group were intraperitoneally injected with 160 μl (5.28 × 10^9^ CFU/mouse), 180 μl (6.0 × 10^9^ CFU/mouse), and 200 μl (6.6 × 10^9^ CFU/mouse) of *S. agalactiae* culture. The clinical manifestations and time of death for mice within each group were observed and recorded over 1 week.

### Immunization Program of BALB/c Mice

**Figure 1:**
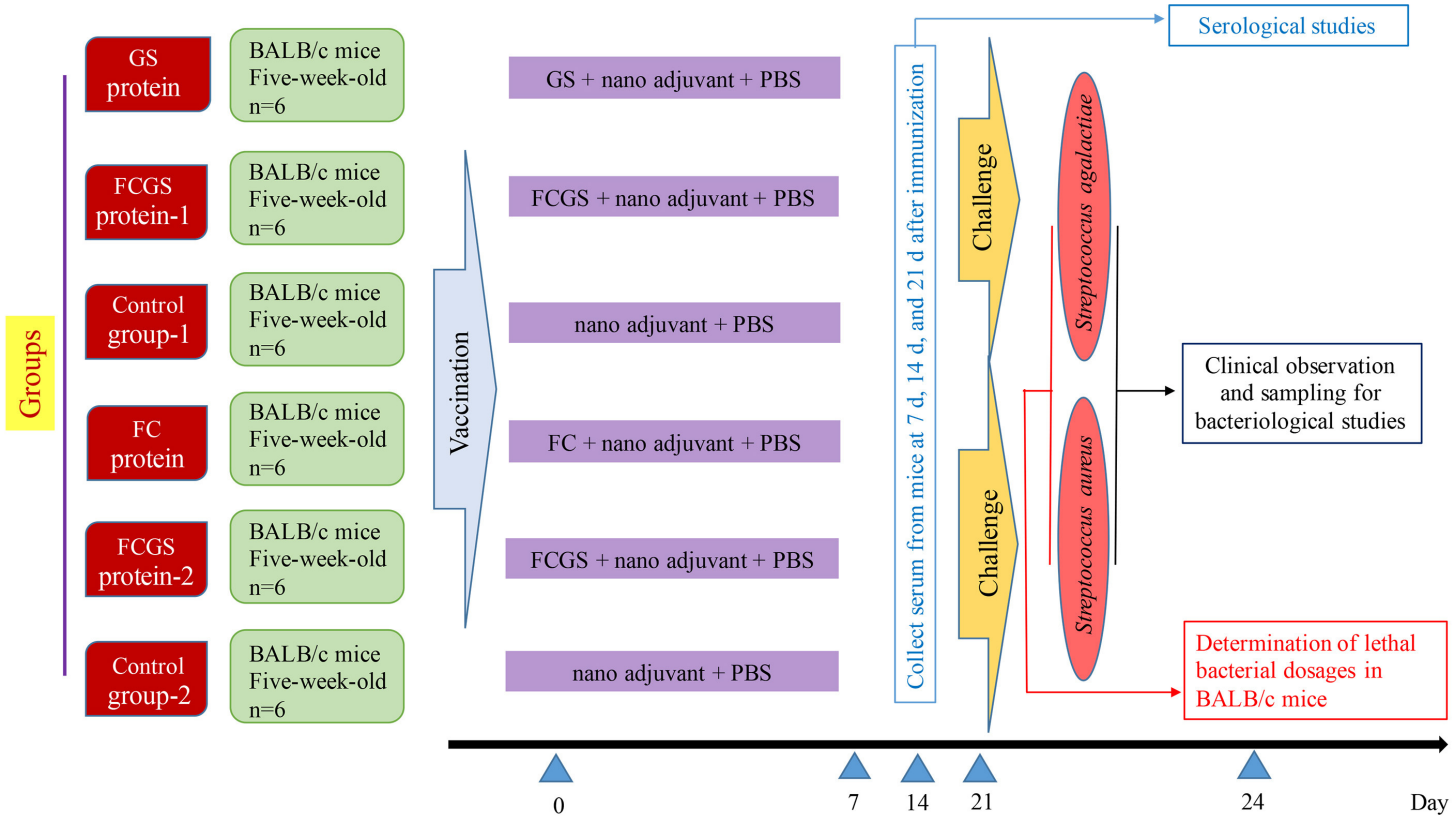
Experimental design and vaccination.

The final concentrations of FC, GS, and FCGS proteins were 4.5, 0.13, and 0.3 mg/ml. The proteins were diluted in PBS and thoroughly mixed with a nano-adjuvant in a 1:1 ratio to obtain FC, GS, and FCGS nano-adjuvant vaccines. We previously screened nano-adjuvants to identify immunological effects of the nano-adjuvants compared with ordinary adjuvants (27). BALB/c mice were randomly divided into six groups, with six mice in each group. The FC, GS, and FCGS nano-adjuvant vaccines were subcutaneously injected (300-μl injections with 40 μg of immunogen per mouse) to immunize mice. PBS and an equal volume of nano-adjuvant were emulsified and injected into mice as a control (Figure 1).

### Indirect ELISA to Detect Antibody Levels

Orbital blood sampling was used to collect serum from mice at 7, 14, and 21 days after immunization. Indirect ELISA was then used to detect IgG antibody levels in mouse sera. ELISA coating solution (Solarbio, China) was used to dilute the antigen to a working concentration; and FC (4.5 ng/ml), GS (2.6 ng/ml), and FCGS (3.0 ng/ml) were added to 96-well plates and kept overnight at 4°C. Each well was washed three times with PBST, 5% skim milk was added, and the plates incubated at 37°C for 2 h. The liquid in each well was then discarded, wells were washed with PBST, and mouse serum (1:2,000) was added and again incubated at 37°C for 1 h. After being washed with PBST, rabbit anti-mouse IgG H&L (HRP) (1:5,000) (Abcam, Cambridge, UK) was added to each well and incubated at 37°C for 1 h. After being washed with PBST, a one-component TMB substrate color developing solution (Solarbio, China) was added, and plates were maintained at room temperature in the dark for 15 min. An ELISA stop solution (Solarbio, China) was then added to each well, and the absorbance at 450 nm (OD value) was measured with a microplate reader within 5 min.

### Challenge Protection Test and Determination of Organ Load

At 21-day post-immunization, *S. aureus* and *S. agalactiae* were cultured to appropriate concentrations, and the mice were intraperitoneally injected with the half-lethal dose. After the challenge, the appearance and mental condition of mice in each group were evaluated, and mouse deaths were recorded.

At 72 h post-bacterial challenge, mice were sacrificed using CO_2_, immersed in 75% ethanol, and dissected under aseptic conditions. Livers and spleens were collected, and the organs were homogenized after adding PBS. Tissue homogenates were diluted to 10^−1^, 10^−2^, and 10^−3^. The homogenates were then spread on BHI Agar (Hopebio, China) and incubated at 37°C for 24–48 h, followed by colony enumeration. Tissue loads were calculated as CFU/g = average number of CFU in the plate × 5 × volume of homogenate (ml) × dilution factor/tissue weight (g). The design and schedule of the study are shown in Figure 1.

### Data Analysis

Statistical significance in differences and correlation coefficients were calculated with SPSS Statistics 23 program. Student's *t*-tests, Student–Newman–Keuls (SNK) tests, and one-way ANOVAs were used to compare measurements among groups. All data are presented as means ± SEM, and data represent the results of three independent experiments. The GraphPad Prism software was used to construct figures.

## Results

### Sequence Selection and Bioinformatics Analysis

Epitope prediction was used as the basis for evaluating protein immunogenicity; and the epitopes of FnBP, ClfA, Sip, and GapC proteins served as the foundation for constructing and expression fusion proteins. The FnBP, ClfA, and GapC did not have signal peptide, while Sip did (20–40 amino acids in length) (Supplementary Figure 1). FnBP had three potential epitopes (propensity index = 0.9725), ClfA had 19 potential epitopes (propensity index = 0.9986), Sip had 19 potential epitopes (propensity index = 1.0315), and GapC had 17 potential epitopes (propensity index = 1.0260) (Supplementary Figure 2). Thus, the four proteins exhibited good immunogenicity. The tertiary structures of recombinant FC, GS, and FCGS were also predicting using Phyre2 (Supplementary Figure 3).

### FC, GS, and FCGS Protein Acquisition

**Figure 2:**
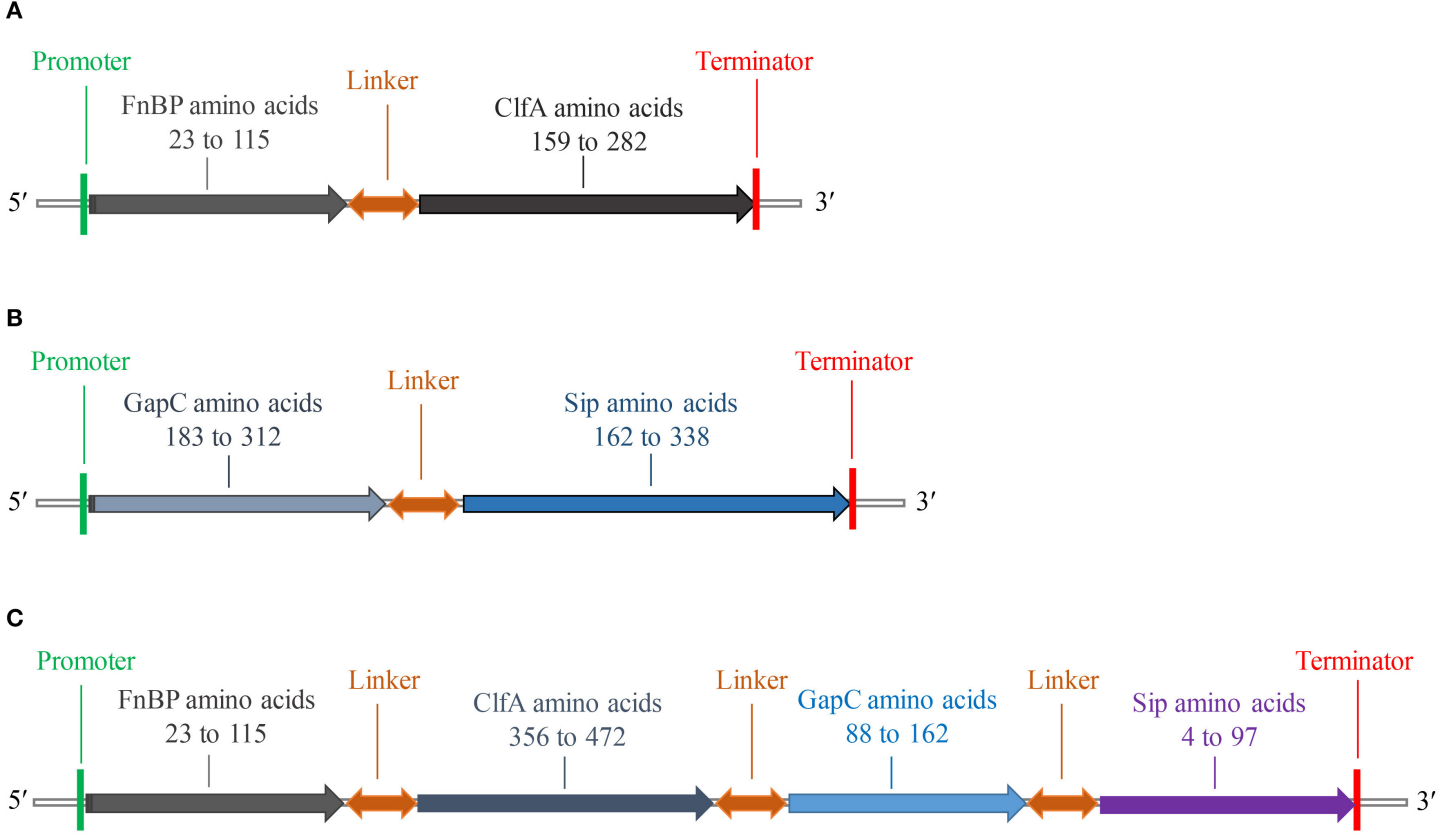
Schematic diagram of FC, GS, and FCGS recombinant proteins. **(A)** *Staphylococcus aureus* FnBP and ClfA (FC) recombinant protein structures. **(B)** *Streptococcus agalactiae* GapC and Sip (GS) recombinant protein structures. **(C)** *S. aureus* FnBP and ClfA, and *S. agalactiae* GapC and Sip (FCGS) recombinant protein structures. Linker sequences (-GGGGSGGGGSGGGGS-) were used as adapters.

The fusion protein of *S. aureus* comprised residues 23–115 of FnBP and 159–282 of ClfA (Figure 2A), while the fusion protein of *S. agalactiae* comprised residues 183–312 of GapC and 162–338 of Sip (Figure 2B). The fusion protein of *S. aureus* and *S. agalactiae* comprised residues 23–115 of FnBP, 356–472 of ClfA, 88–162 of GapC, and 4–97 of Sip (Figure 2C). The amino acid sequences of FC, GS, and FCGS are shown in Supplementary Figure 4.

**Figure 3:**
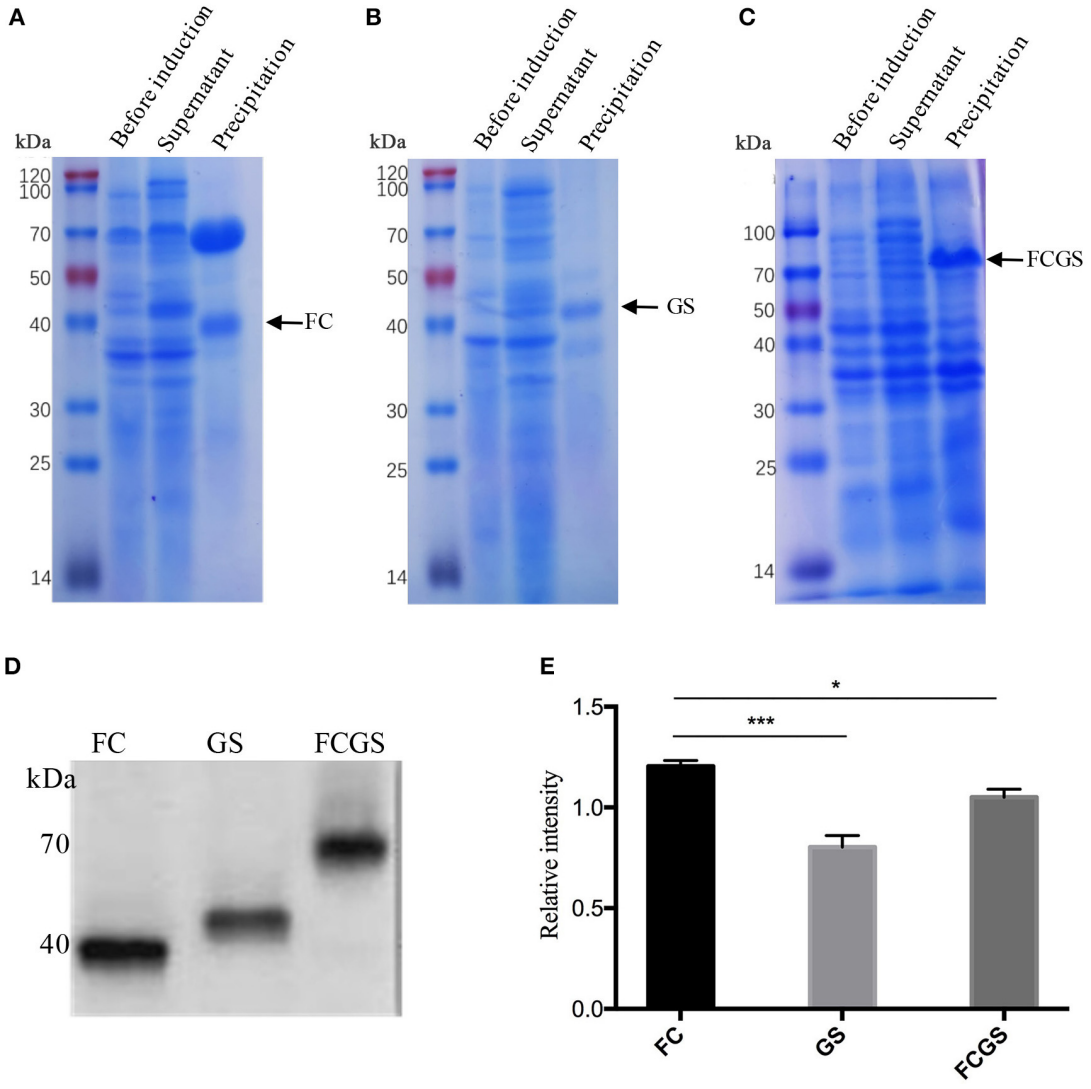
FC, GS, and FCGS recombinant protein identification and characterization. **(A)** Expression of FC recombinant fusion protein in *Escherichia coli*. **(B)** Expression of GS recombinant fusion protein in *E. coli*. **(C)** Expression of FCGS recombinant fusion protein in *E. coli*. IPTG was used to induce recombinant expression of pET32a-FC-DE3, pET28a-GS-DE3, and pET32a-FCGS-DE3 for 6 h; cells were collected by centrifugation and then subjected to sodium dodecyl sulfate–polyacrylamide gel electrophoresis (SDS-PAGE) analysis. **(D)** Western blotting identification of purified FC, GS, and FCGS recombinant proteins. **(E)** Relative intensities of FC, GS, and FCGS were semi-quantified using ImageJ software program. Data show means ± SEM, *p < 0.05 and ***p < 0.001. Results were obtained from three independent replicate experiments.

The recombinant plasmids of each expression vector were transferred into *E. coli* DE3 competent cells, resulting in the expression of the FC (pET32a-FC) (Figure 3A), GS (pET28a-GS) (Figure 3B), and FCGS (pET32a-FCGS) fusion proteins (Figure 3C). SDS-PAGE analysis indicated that the FCGS, FC, and GS recombinant proteins were all expressed in bacterial inclusion bodies (Figures 3A–C). Post-purification, the purity of FC, GS, and FCGS was >90%, >85%, and >85%, respectively (Supplementary Figure 5). Western blotting analysis confirmed that the target proteins reacted with the positive sera of *S. aureus* and *S. agalactiae* and had good reactogenicity (Figure 3D). A semi-quantitative analysis of Western blotting bands was also conducted (Figure 3E).

### Determination of Lethal Doses in BALB/c Mice

**Figure 4:**
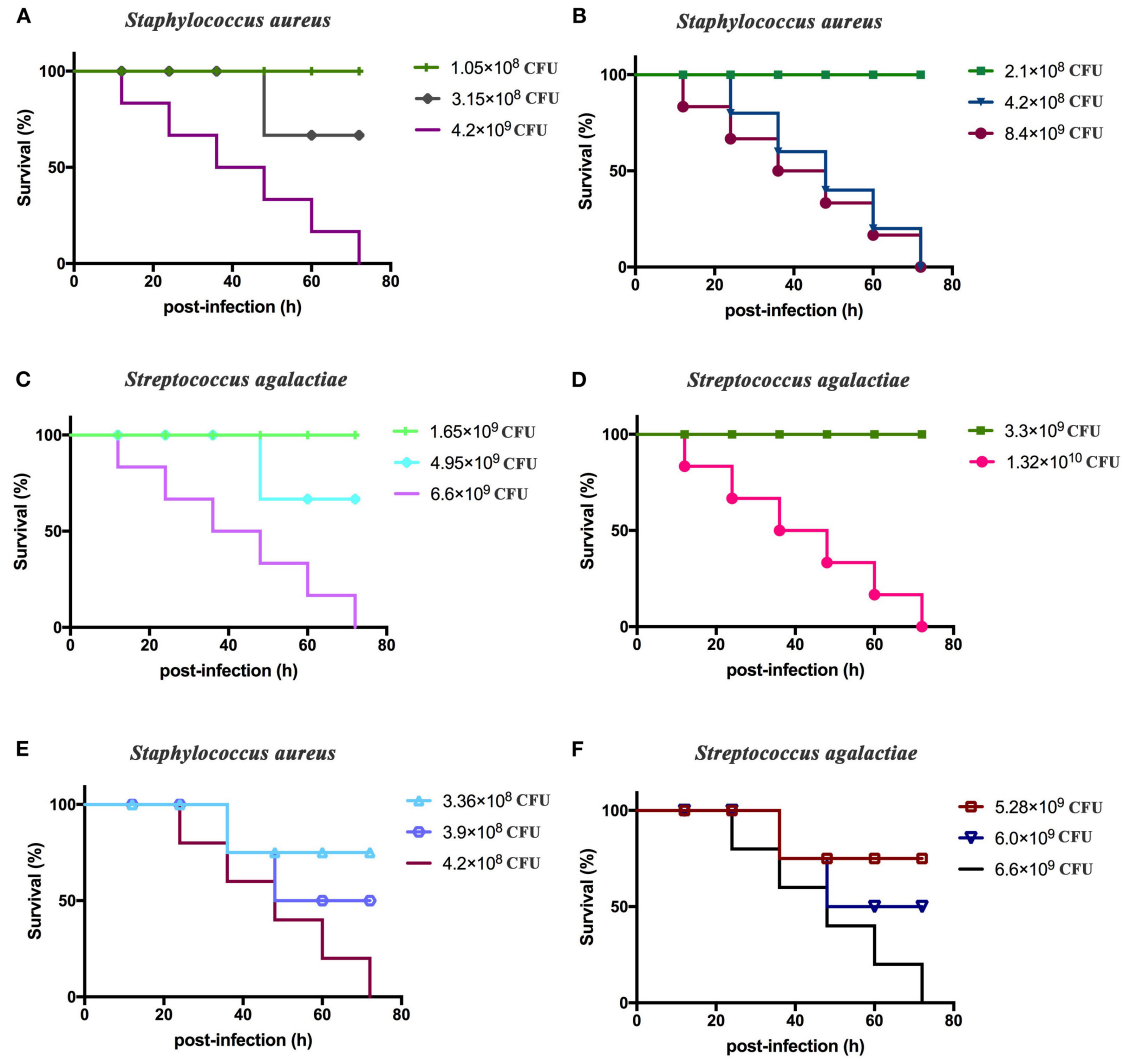
*Staphylococcus aureus* and *Streptococcus agalactiae* total and half lethal doses in BALB/c mouse challenges. After *S. aureus* and *S. agalactiae* cells were collected by centrifugation, BALB/c mice were immunized based on CFU/mouse dosages in the analyses; and mouse survival was recorded over 72 h. **(A,B)** The total lethal dose of *S. aureus* to BALB/c mice. **(C,D)** The total lethal dose of *S. agalactiae* to BALB/c mice. **(E)** The half lethal dose of *S. aureus* to BALBC/c mice; **(F)** the half lethal dose of *S. agalactiae* to BALB/c mice. The results represent three independent replicate experiments.

BALB/c mice displayed clinical symptoms within 12 h after intraperitoneal injection of *S. aureus* and *S. agalactiae*. Initial manifestations included a lack of energy, slower movements, reduced eating, reduced drinking, curling up in a corner, and rapid breathing. The mice did not die after 72 h (Figure 4). The total lethal doses were 4.2 × 10^8^ CFU/mouse for *S. aureus* and 6.6 × 10^9^ CFU/mouse for *S. agalactiae* (Figures 4A–D). For the LD50 tests, mice showed clinical manifestations similar to total lethal doses, but the symptoms were alleviated, and they were able to crawl slightly. After 72 h, LD50 values were 3.9 × 10^8^ CFU for *S. aureus* and 6.0 × 10^9^ CFU for *S. agalactiae* (Figures 4E,F).

### Levels of Antibodies Induced by FC, GS, and FCGS Recombinant Proteins

**Figure 5:**
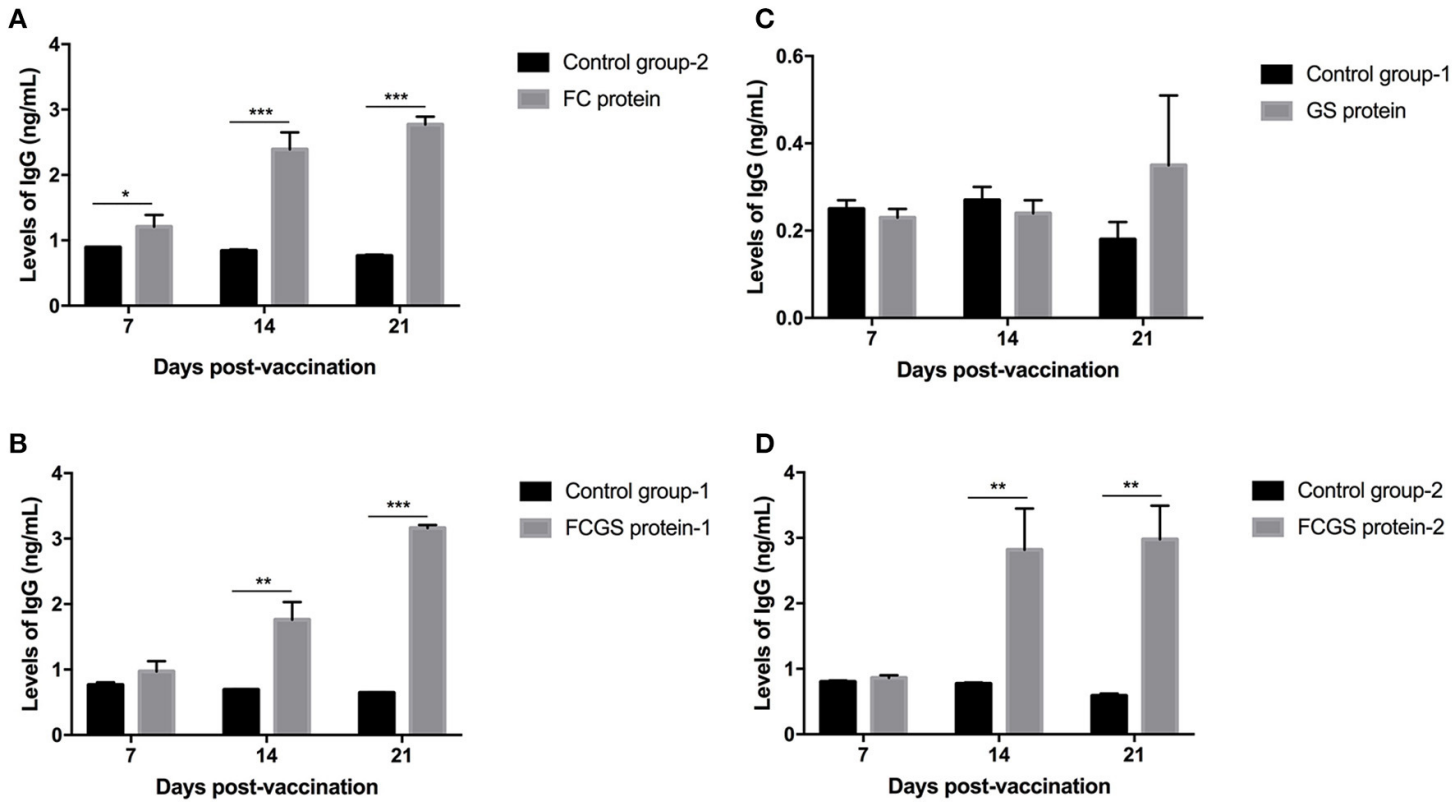
Levels of IgG antibodies induced by FC, GS, and FCGS recombinant proteins in BALB/c mice. Mice were immunized with protein and adjuvants, followed by collection of mouse sera at 7, 14, and 21 days post-immunization to detect IgG content with ELISA. **(A)** The specific IgG levels in the serum of mice at different time periods after FC immunization. **(B,D)** The specific IgG levels in the serum of mice at different time periods after FCGS immunization. **(C)** The specific IgG levels in the serum of mice at different time periods after GS immunization. Data are means ± SEM (*p < 0.05, **p < 0.01, ***p < 0.001). The results were obtained from three independent replicate experiments.

The procedure shown in Figure 1 was followed for the sequential study. FC, GS, and FCGS fusion proteins were used to coat the ELISA plates to detect the production of antibodies in mice immunized with the three proteins. On the 14th and 21st days post-immunization, antibody levels in mice induced by the FC recombinant protein were significantly higher than in the control group (*p* < 0.001) (Figure 5A). It is worth noting that the FC fusion protein rapidly induced antibody production in mice on the seventh day (*p* < 0.05) (Figure 5A). The FCGS fusion protein-1 group produced significantly higher antibody levels, higher than those of the control group on the 14th day after immunization (*p* < 0.01), and the difference was extremely significant on the 21st day (Figure 5B). The GS fusion protein induced the mice to produce higher antibody levels than the control group on the 21st day after immunization, but the difference was not significant (Figure 5C). In addition, compared with the control group on the 14th and 21st days after immunization, the FCGS protein-2 group had significant difference (*p* < 0.01) (Figure 5D). Thus, the antibody levels induced by the FCGS fusion protein were higher than those due to the FC and GS recombinant proteins.

### Analysis of the Protective Effects of FC, GS, and FCGS Recombinant Proteins

Standard *S. aureus* and *S. agalactiae* strains were used to challenge mice 21 days after immunization. The FC protein conferred good resistance to *S. aureus*, and the GS protein was effective against *S. agalactiae*. Significant resistance was also conferred by FCGS protein to *S. agalactiae* and *S. aureus* (Table 1).

**Table 1:** Mouse challenge immune protection results.

**Groups**	**Death/survival**	**Protection rate**
FC protein	2/4	66.7%
GS protein	2/4	66.7%
FCGS protein-1	2/4	66.7%
FCGS protein-2	2/4	66.7%
Control group-1	3/3	50%
Control group-2	3/3	50%

**Figure 6:**
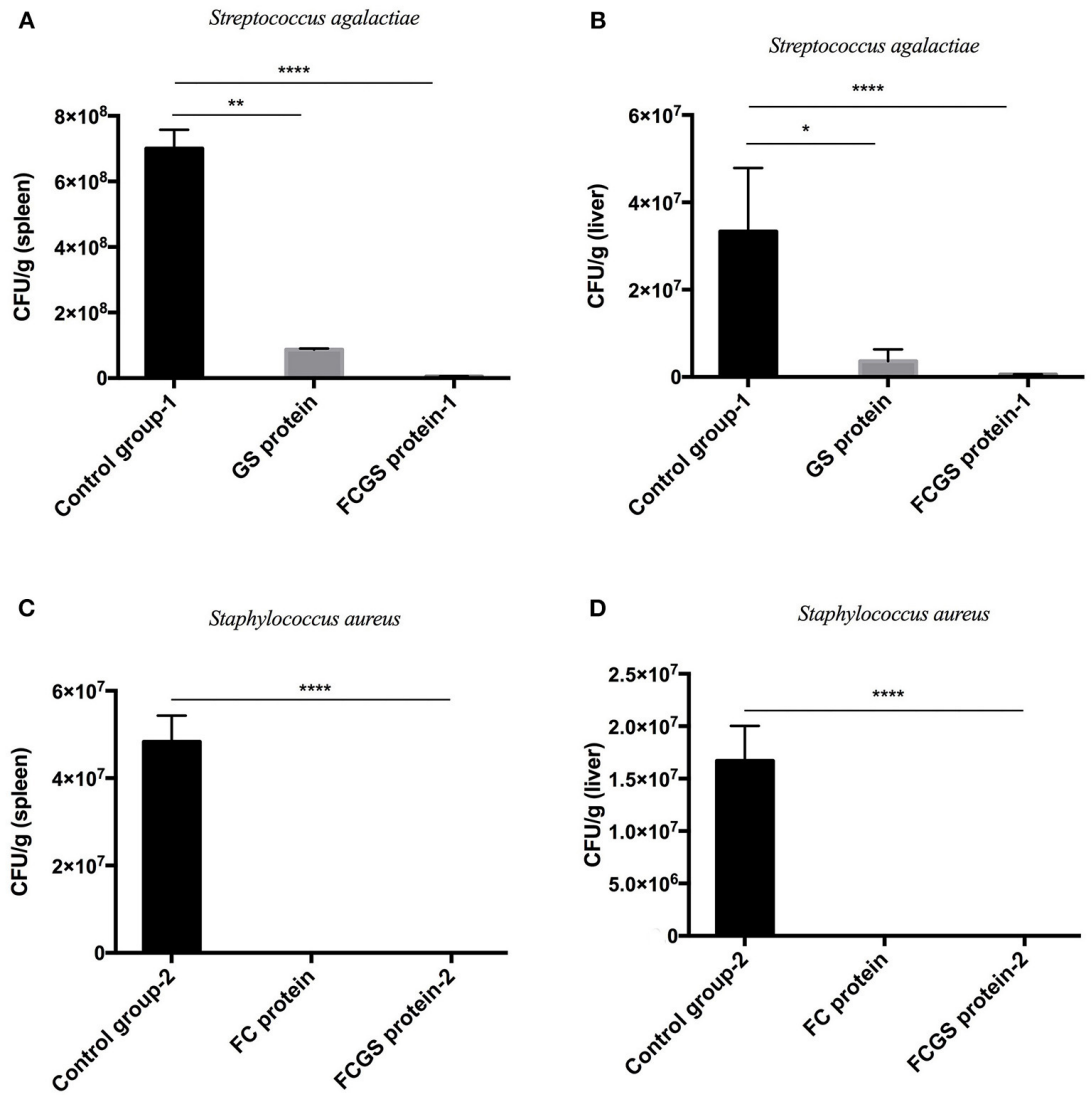
Bacterial loads in the spleens and livers of BALB/c mice after *Staphylococcus aureus* and *Streptococcus agalactiae* challenges. At 21 days after immunization with FC, GS, and FCGS proteins, mice were challenged with *S. aureus* and *S. agalactiae*. The spleens and livers of the mice were aseptically collected, homogenized, and spread on plates to count colonies. **(A,B)** After the challenge of *S. agalactiae*, the bacterial loads in the organs of the mice in the GS and FCGS groups are shown. **(C,D)** After the challenge of *S. aureus*, the bacterial loads in the organs of the mice in the FC and FCGS groups are shown. Data are shown as means ± SEM (*p < 0.05, **p < 0.01, ****p < 0.0001). Results represent three independent replicate experiments.

At 72 h post-bacterial challenge, the bacterial loads in the spleens and livers were enumerated. Mice immunized with the FCGS and GS recombinant proteins withstood challenges with *S. agalactiae*, as indicated by fewer bacteria isolates compared with the control group (Figures 6A,B). The FCGS and FC recombinant proteins also conferred resistance to *S. aureus* after immunization compared with the control group, as evinced by significantly lower numbers of isolated colonies (Figures 6C,D). These results were consistent with FC, GS, and FCGS recombinant protein antibody levels (Figure 5) and challenge protection efficiency (Table 1), indicating that the FC, GS, and FCGS recombinant proteins can cause specific immune responses in mice. Thus, FC immunization was effective against *S. aureus*, and GS immunization was effective against *S. agalactiae* infection, while FCGS immunization was effective toward challenges of both *S. aureus* and *S. agalactiae*.

## Discussion

Bioinformatics analysis, comparison of protein-associated information, and predictions of epitopes have become important methods in immunological research (28). Here, a signal peptide was observed at the N-terminus of the Sip protein, indicating that it is exported outside of the cell and may be related to bacterial cell wall function. Nevertheless, the mechanism mediating the association with cell walls has not been determined. FnBP, ClfA, GapC, and Sip proteins also were observed to exhibit multiple B cell epitopes, indicating that they can act as antigens to initiate immune response. Predictive analysis was used here to fuse the four genes of *S. aureus* FnBP and ClfA in addition to *S. agalactiae* GapC and Sip, thereby overcoming the low level of specific immunity induced by a single gene. Indeed, the recombinant fusion proteins FC, GS, and FCGS could stimulate the production of an immune response in mice. Further, immunizing mice with these recombinant proteins generated resistance to challenges with *S. aureus* and *S. agalactiae*. Thus, the FC, GS, and FCGS recombinant proteins identified here have potential value in mastitis vaccine development.

Adhesion is the first step during biofilm formation by pathogens or the invasion of host cells. Adhesion also protects bacteria from the host's immune system and promotes chronic infection. The primary mechanism by which *S. aureus* adheres to cells and invades non-specific phagocytes (FnBP-Fn-α5β1 integrin) is critical to target in the effective treatment of chronic staphylococcal infections (29). Castagliuolo et al. (30) used a mixture of pDNA encoding four adhesion hormones (ClfA, FnBPA, EfB, and Can) to intranasally immunize mice, with the treatment effectively reducing IMI caused by *S. aureus*. Delfani et al. (31) used a ClfA-ISDB-HLG recombinant protein to immunize BALB/C mice, wherein antibodies induced by the protein could promote enhanced phagocytosis of *S. aureus* by macrophages. Hall et al. (32) passively immunized mice with ClfA monoclonal antibodies, observing that this approach could prevent mastitis caused by *S. aureus* in mice. In addition, Song et al. (33) intramuscularly injected mice with XL1-Blue/LOG76 or XL1-Blue/LO11 strains in addition to recombinant GapC_1−150_. The immunized mice then resisted challenge with *Streptococcus* strains. Thus, the use of GapC_1−150_ on the surface of *E. coli* is a feasible vaccine approach against *Streptococcus* infection. El-Din et al. (34) immunized dairy cows with a DNA vaccine expressing ClfA plasmids, and the cows produced a strong specific antibody response against ClfA. Other studies have indicated that the Fc-Sip+Fc-FnBPB-ClfA dual subunit vaccine exhibits better therapeutic and preventive effects against *S. agalactiae* and *S. aureus* mastitis in dairy cows (35). Consequently, new anti-staphylococcal drugs are suitable vaccine agents and have been proposed based on structural models of ClfA–Fg interactions (36).

GapC chimeric proteins constructed from the non-conservative peptide regions of GapC from *S. agalactiae* and *Shigella dysenteriae* have been shown to retain the characteristics of the wild-type GapC protein of *Streptococcus uberis* (20). Further, other studies have reported that *E. coli* expression of recombinant Sips of *Streptococcus* can improve humoral immunity, while purified rSip can better induce the IgG anti-Sip immune responses and play an active role in reducing group B streptococcus (GBS) vaginal colonization (37). Chimeric CAMP (CAMP-3) of *S. uberis* and *S. agalactiae* can produce immune cross-reactive vaccine antigens that are more effective than any CAMP factor alone. In addition, inoculation of *S. uberis* several days after challenge can significantly reduce inflammation and provide protection (21). Herein, FC, GS, and FCGS recombinant proteins were obtained by expression and purification in bacteria, and their effective reactogenicity was confirmed by Western blotting analysis. Mice immunized with recombinant proteins were protected from bacterial irritation. Likewise, purified CP+Sip-FbsA conjugate mixed with aluminum salt adjuvant exhibited a stronger immunoprotective effect on Wistar rats infected with *S. agalactiae* during lactation (38). Importantly, the Sip subunit vaccine of *S. agalactiae* promotes the occurrence of humoral and local immune response in dairy cows and can reduce the number of somatic cells in milk (39).

The screening of the protective antigens from *S. aureus* including FnBP and ClfA in addition to *S. agalactiae* GapC and Sip provides a framework for the combined use of antigens with other virulence factors in the development of *S. aureus* and *S. agalactiae* vaccines. In particular, the FC, GS, and FCGS fusion proteins developed here have the potential to be used as vaccines against *S. aureus* or *S. agalactiae* infections. Among these, the FCGS fusion protein has the ability to simultaneously resist *S. aureus* and *S. agalactiae* infections, indicating that it is an important target for future studies. Both the FC and FCGS fusion proteins represent the Fnbp and ClfA genes, although the selected target fragments differ, implying that the same gene comprises different specific antigen fragments, and different combinations may alter the immune protection of recombinant proteins. It should also be noted that the results of this study are based on a limited number of mice, and although representative data were generated, a larger experimental population or an experiment with dairy cows is needed to verify the effectiveness of vaccines identified here.

## Conclusion

FC, GS, and FCGS recombinant fusion proteins induced BALB/c mice to produce specific antibodies and provided effective protection against *S. aureus* and *S. agalactiae* infections. In particular, FCGS proteins conferred resistance to *S. aureus* and *S. agalactiae* and should be a key vaccine focus for future studies to mitigate mastitis.

## Data Availability Statement

The raw data supporting the conclusions of this article will be made available by the authors, without undue reservation.

## Ethics Statement

The animal study was reviewed and approved by Animal Care and Use Committee of Shihezi University.

## Author Contributions

ZM, XY, and PW designed the study. ZM and PW were responsible for the development, integration, and writing of the manuscript. PW and CC reviewed the article. RH, YW, JY, and ZW provided help during the experiments. All authors contributed to the article and approved the submitted version.

## Conflict of Interest

The authors declare that the research was conducted in the absence of any commercial or financial relationships that could be construed as a potential conflict of interest.

## Supplementary Material

Supplementary Figure 1 FnBP, ClfA, GapC, and Sip protein signal peptide predictions. **(A)** FnBP protein signal peptide prediction. **(B)** ClfA protein signal peptide prediction. **(C)** Sip protein signal peptide prediction. **(D)** GapC protein signal peptide prediction. The predictions were generated with the SignalP 5.0 server (http://www.cbs.dtu.dk/services/SignalP/).

Supplementary Figure 2 FnBP, ClfA, GapC, and Sip protein B cell epitope predictions. **(A)** FnBP protein B cell epitope prediction. **(B)** ClfA protein B cell epitope prediction. **(C)** Sip protein B cell epitope prediction. **(D)** GapC protein B cell epitope prediction. The predictions were generated from http://imed.med.ucm.es/Tools/antigenic.pl.

Supplementary Figure 3 Three-dimensional structure prediction for FC, GS, and FCGS proteins. **(A)** FC protein structure prediction. **(B)** GS protein structure prediction. **(C)** FCGS protein structure prediction. The structures were generated with Phyre2 (http://www.sbg.bio.ic.ac.uk/phyre2/html/page.cgi?id=index).

Supplementary Figure 4 Amino acid sequences of FC, GS, and FCGS proteins. **(A)** FC protein amino acid sequences. **(B)** GS protein amino acid sequences. **(C)** FCGS protein amino acid sequences.

Supplementary Figure 5 Purity identification of FC, GS, and FCGS proteins. The purified proteins was analyzed by SDS-PAGE to observe its purity. The pictures represent the results of three independent trials.
