# Supplementary figures and images for: The Recombinant Expression Proteins FnBP and ClfA From Staphylococcus aureus in Addition to GapC and Sip From Streptococcus agalactiae Can Protect BALB/c Mice From Bacterial Infection
Source: Front Vet Sci. 2021 Jun 24;8:666098. doi: 10.3389/fvets.2021.666098 (PMC8263938; doi:10.3389/fvets.2021.666098)

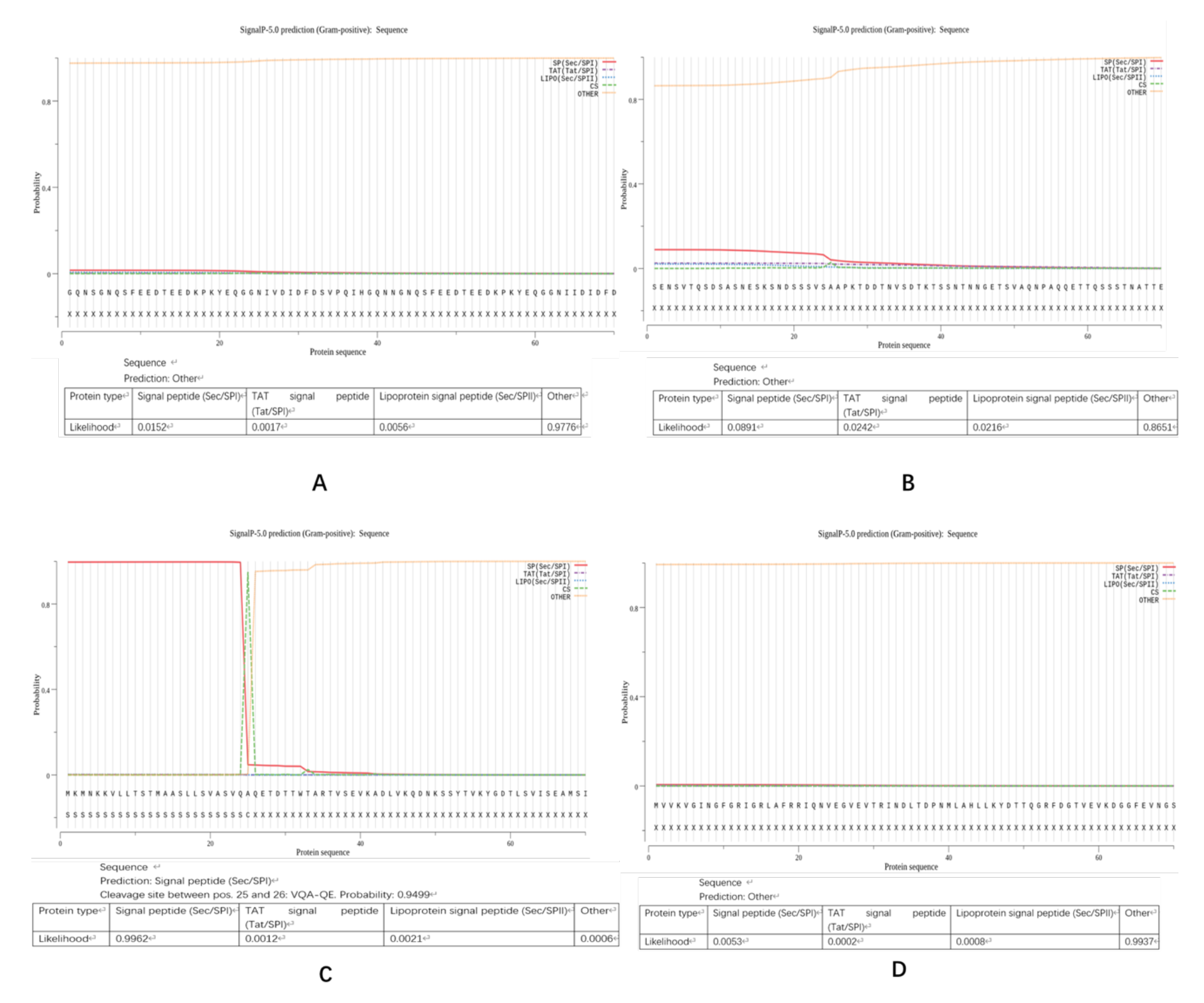

Supplement: Supplementary Figure 1 — FnBP, ClfA, GapC, and Sip protein signal peptide predictions. (A) FnBP protein signal peptide prediction. (B) ClfA protein signal peptide prediction. (C) Sip protein signal peptide prediction. (D) GapC protein signal peptide prediction. The predictions were generated with the SignalP 5.0 server (http://www.cbs.dtu.dk/services/SignalP/). [file Image_1.TIF]

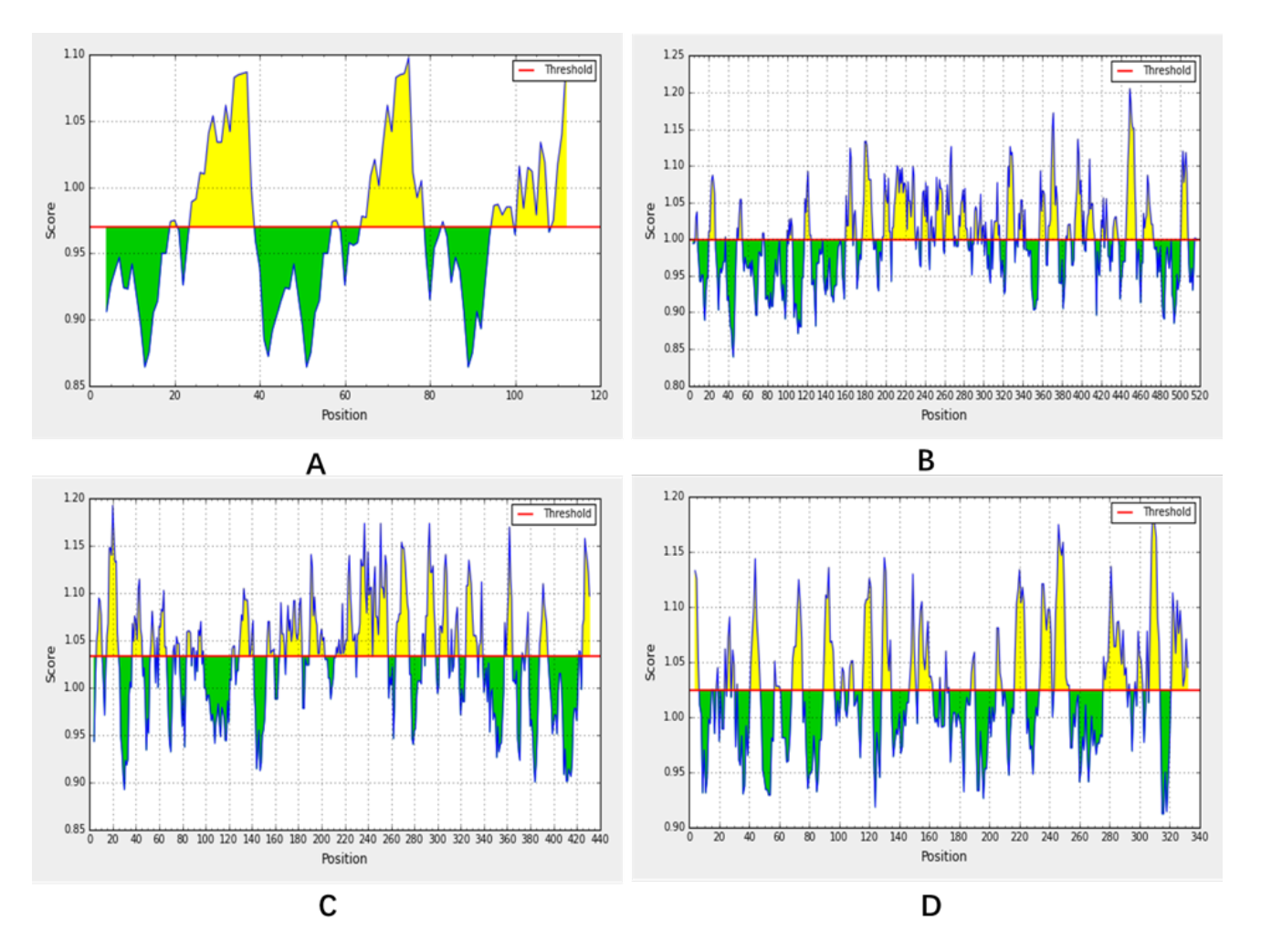

Supplement: Supplementary Figure 2 — FnBP, ClfA, GapC, and Sip protein B cell epitope predictions. (A) FnBP protein B cell epitope prediction. (B) ClfA protein B cell epitope prediction. (C) Sip protein B cell epitope prediction. (D) GapC protein B cell epitope prediction. The predictions were generated from http://imed.med.ucm.es/Tools/antigenic.pl. [file Image_2.TIF]

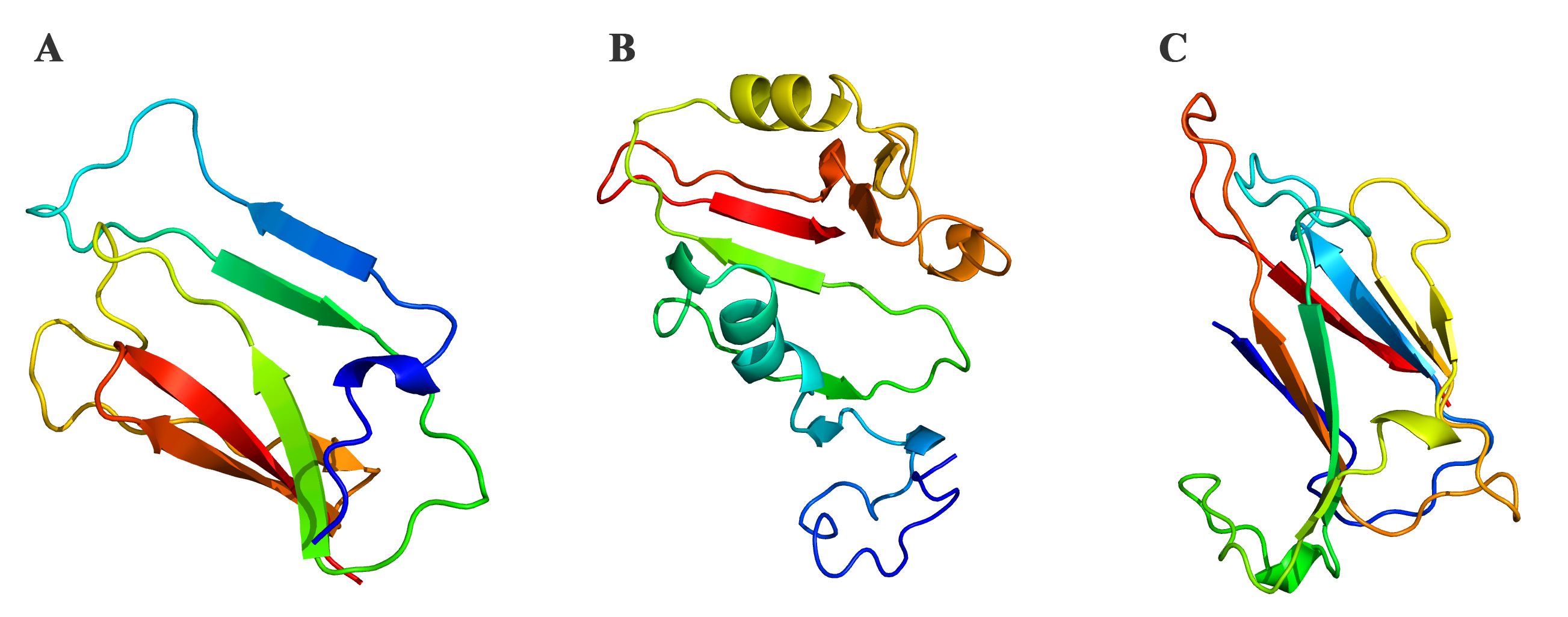

Supplement: Supplementary Figure 3 — Three-dimensional structure prediction for FC, GS, and FCGS proteins. (A) FC protein structure prediction. (B) GS protein structure prediction. (C) FCGS protein structure prediction. The structures were generated with Phyre2 (http://www.sbg.bio.ic.ac.uk/phyre2/html/page.cgi?id=index). [file Image_3.JPEG]

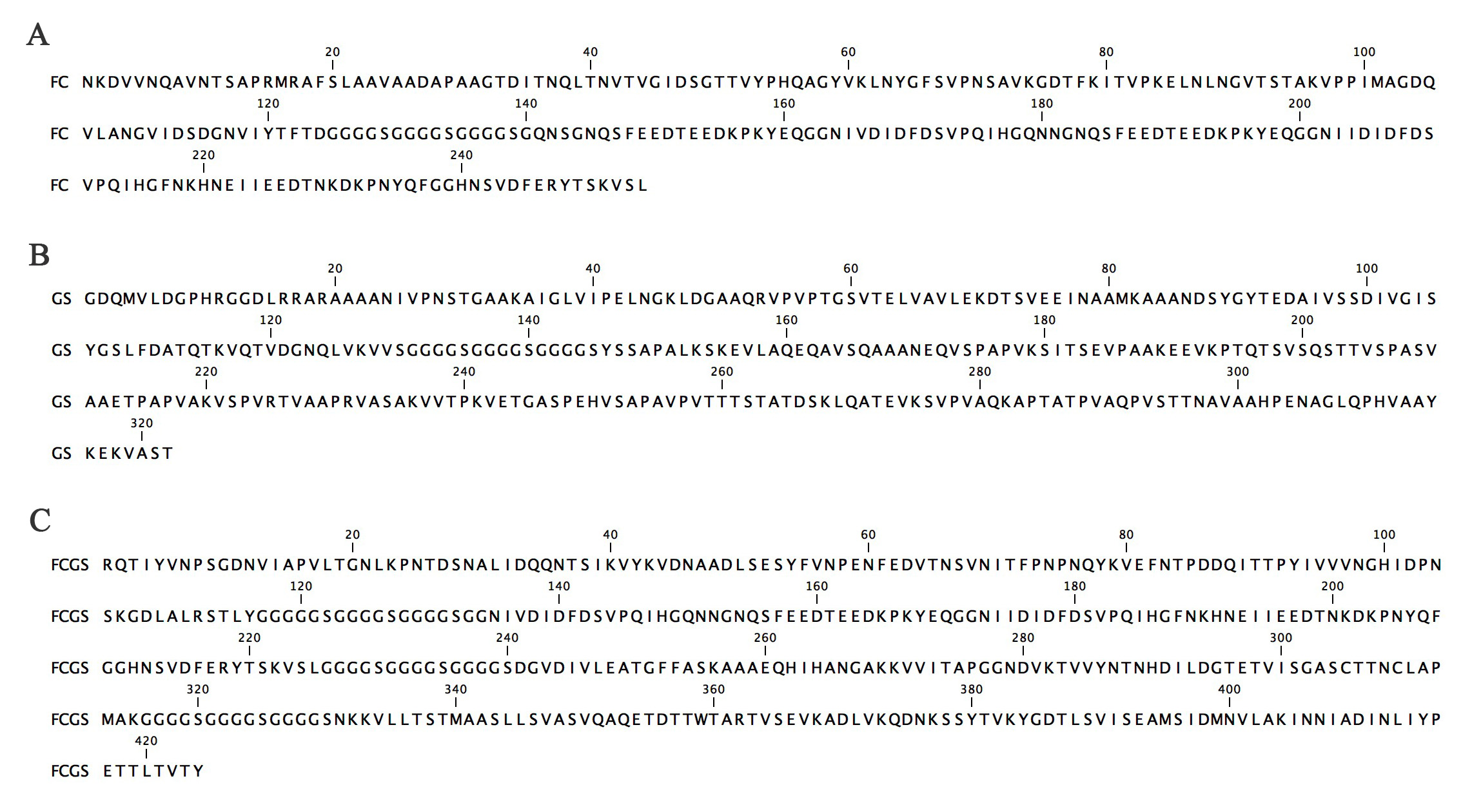

Supplement: Supplementary Figure 4 — Amino acid sequences of FC, GS, and FCGS proteins. (A) FC protein amino acid sequences. (B) GS protein amino acid sequences. (C) FCGS protein amino acid sequences. [file Image_4.JPEG]

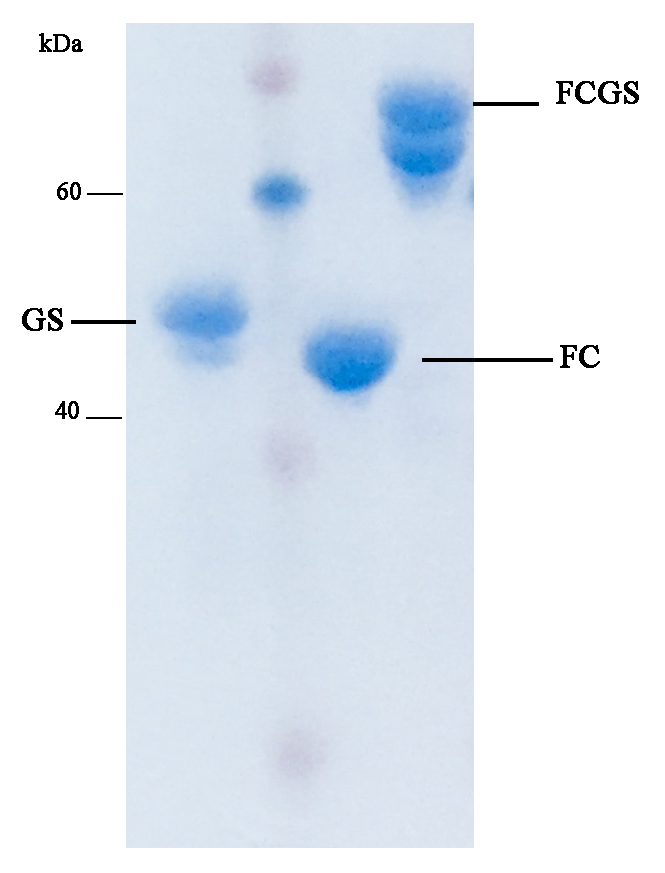

Supplement: Supplementary Figure 5 — Purity identification of FC, GS, and FCGS proteins. The purified proteins was analyzed by SDS-PAGE to observe its purity. The pictures represent the results of three independent trials. [file Image_5.JPEG]
